# Over-the-Scope Clip Applications as First-Line Therapy in the Treatment of Upper Non-variceal Gastrointestinal Bleeding, Perforations, and Fistulas

**DOI:** 10.3389/fmed.2022.753956

**Published:** 2022-02-15

**Authors:** Jiayu Qiu, Jun Xu, Yanxia Zhang, Foqiang Liao, Zhenhua Zhu, Xu Shu, Youxiang Chen, Xiaolin Pan

**Affiliations:** ^1^Department of Gastroenterology, The First Affiliated Hospital of Nanchang University, Nanchang, China; ^2^Human Genetic Resources Center, The First Affiliated Hospital of Nanchang University, Nanchang, China

**Keywords:** OTSC (over-the-scope clip), endoscopy, first-line therapy, upper non-variceal gastrointestinal bleeding (UNVGIB), perforation, fistula

## Abstract

**Background:**

The over-the-scope clip (OTSC) is an innovative device and has been successfully used in endoscopic treatment, however, there is a lack of clinical data from China. The aim of this study is to investigate the OTSC applications in the treatment of upper non-variceal gastrointestinal bleeding (UNVGIB), perforations, and fistulas in China.

**Methods:**

In total, 80 patients were treated with one OTSC respectively as first-line therapy in our endoscopy center between January 2016 and November 2020. Among them, 41 patients had UNVGIB, 34 patients had perforations, and five patients had fistulas. The technical and clinical success rates were used to assess the efficacy of OTSC on the above diseases. In addition, we compared the hemostatic efficacy of OTSC with the standard endoscopic therapy in ulcer bleeding and Dieulafoy's lesion by propensity score matching analysis.

**Results:**

In general, the OTSCs were applied successfully in all patients and achieved 100% (80/80) technical success. The clinical success of all patients was 91.3% (73/80). Among 41 patients with UNVGIB, the clinical success was 85.4% (35/41); 6 patients presented with recurrence. For patients of Dieulafoy's lesion and under antithrombotic therapy, we found that OTSC treatment had both efficient and reliable hemostasis effects. In addition, according to the characteristics of ulcers, site of bleeding lesion, and Blatchford score, all patients received similar and reliable clinical success rates. After propensity score matching, we found that OTSC treatment had low rebleeding rates when compared with standard endoscopic therapy in both Dieulafoy's lesion (15.0 vs 30.0%) and ulcer bleeding (17.6 vs 29.4%). Among 34 patients with perforations, the clinical success was 100% (34/34). Among five patients with fistulas, only one patient failed in maintaining the OTSC before esophageal fistula healing, and the clip achieved an overall clinical success of 80% (4/5).

**Conclusion:**

The OTSC represents a safe and effective endoscopic therapy for UNVGIB, perforations, and fistulas as first-line treatment, especially for Dieulafoy's lesion or patients under antithrombotic therapy for UNVGIB, etc. However, OTSC application in these specific lesions or patients lacks adequate evidence as first-line treatment. Therefore, further larger sample and multi-center clinical trials are required to improve its indications in clinical treatment.

## Introduction

With the advancement of endoscopic techniques, many gastrointestinal lesions have been properly managed, but there are still challenges. Most upper non-variceal gastrointestinal bleeding (UNVGIB) could be treated by conventional endoscopic therapies, but recent studies have shown that some UNVGIB such as Dieulafoy's lesion and refractory bleeding could not be treated successfully and develop recurrence easily after standard endoscopic therapies such as epinephrine injections, hemoclips, or coagulation (1, 2). Equally, although many treatments have been attempted for the closure of perforations and fistulas, more and more iatrogenic gastric perforations are raised during endoscopic resection such as endoscopic mucosal resection (EMR) and endoscopic submucosal dissection (ESD), which could be handled by endoscopic closure and conservative management. However, there are still rare cases that need surgery (3, 4). Moreover, the closure of gastrointestinal fistulas is currently difficult because of the fibrosis tissue, inadequate opening width, and so on (5).

The over-the-scope clip (OTSC; OVESCO Endoscopy AG, Tuebingen, Germany), an innovative endoscopic full-thickness suturing device, has been developed and spread worldwide since it was firstly introduced for the closure of iatrogenic colon perforations in an animal experiment in 2007 (6). After that, in 2008, its effective application for UNVGIB and perforations in humans was confirmed in clinical experiences (7). In subsequent experimental studies, its indications have been further evaluated in many ways such as the closure of transgastric natural orifice transluminal endoscopic surgery (NOTES), perforations, fistulas, hemorrhage, and marking lesions before surgery in the gastrointestinal tract (8–14). For UNVGIB, the use of OTSC has emerged in recurrent ulcer bleeding treatment with promising results (15–17). However, data on use of OTSC as first-line therapy are very limited (17, 18). To date, the evidence on the efficacy of OTSC in Dieulafoy's lesion is derived from case series or small descriptive studies, meanwhile, studies on the comparison with standard endoscopic therapy are lacking (16, 18).

The OTSC was academically promoted officially in China and has become popular since 2014. However, the studies on OTSC application are mainly from European countries, and the published clinical data from China are still lacking (13). Here, we analyze and present the retrospective clinical study results of 80 patients, using the OTSC system for UNVGIB, perforations, and fistulas in a tertiary care hospital of China. Meanwhile, we investigate comparative outcomes of OTSC as first-line therapy versus standard endoscopic therapy in ulcer bleeding and Dieulafoy's lesion by using propensity score matching analysis.

## Materials and Methods

### Patient Selection

**Figure 1:**
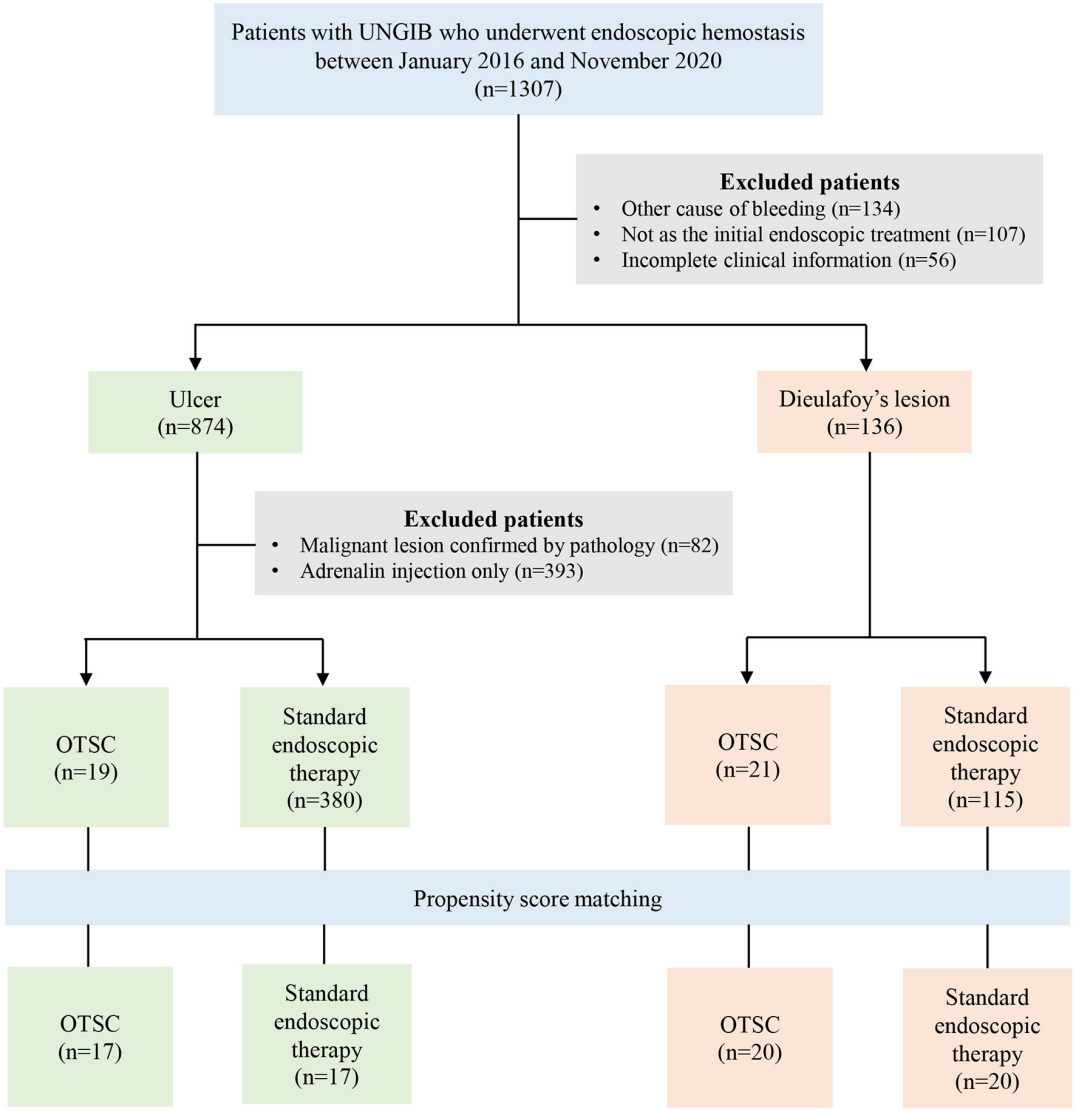
A flowchart of the study cohort in patients with UNVGIB.

We conducted a single-center retrospective study on the application of OTSC as first-line treatment in patients who were confirmed by endoscopy to have UNVGIB, perforations, and fistulas. A total of 80 patients underwent OTSC placement between January 2016 and November 2020 in our Endoscopy Center and each patient was treated with only one OTSC as first-line treatment respectively. To assess the efficiency of OTSC application as first-line treatment in UNVGIB (including large, fibrotic ulcer beds with obvious visible vessel or ulcers where the endoscopic treatment was difficult to perform, which may not be amenable to conventional endoscopic therapies, Dieulafoy's lesion, and other cause of UNVGIB), 41 patients were screened for eligibility. In addition, to further investigate the efficiency of OTSC application compared with the standard endoscopic therapy in ulcer bleeding and Dieulafoy's lesion of UNVGIB by propensity score matching analysis, 1,307 consecutive patients with UNVGIB who underwent endoscopic hemostasis were screened for eligibility at the same period. The inclusion criteria were as follows: (a) Patients with UNVGIB. (b) Patients treated with endoscopic hemostasis. The exclusion criteria were as follows: (a) Other cause of bleeding than ulcer bleeding and Dieulafoy's lesion. (b) This endoscopic therapy was not the initial treatment. (c) Patients with incomplete clinical information. (d) Patients with malignant lesions confirmed by pathology. (e) Endoscopic hemostasis with only epinephrine injection (endoscopic monotherapy of epinephrine injection is not recommended by recent guidelines) (15). Among them, they were divided into ulcer bleeding and Dieulafoy's lesion according to the lesion type. Each type was further divided into “OTSC” group and “standard endoscopic therapy” group according to whether OTSC was used or not. The flowchart of patient selection is shown in Figure 1. Meanwhile, to evaluate the efficiency of OTSC application as first-line treatment in perforations and fistulas, 34 patients with perforation and 5 patients with fistula were screened for eligibility. The inclusion criteria for perforations and fistulas were as follows: (a) Patients with perforations or fistulas. (b) Patients treated with OTSC as first-line treatment. The study was approved by the Ethics Committee of the First Affiliated Hospital of Nanchang University.

The informed consent of patients was obtained before all endoscopic treatment. The medical history of patients was collected and analyzed including their demographics, indications for OTSC interventions, the characteristic of lesions, recurrence of the original lesion, as well as the technical and clinical success. The definition of technical success is the successful application at the targeted lesion when using OTSC. Clinical success is known as the achievement of the intent outcome without the need for additional therapies within 30 days during the follow-up. Recurrence was defined by the repeated symptoms of bleeding, perforation, or fistula after endoscopic treatment during the 30 days of follow-up (11, 19).

### The OTSC System

The OTSC system consists of an over-the-scope clip, an applicator cap, a hand wheel, and a twin or anchor type grasper. The clip is mounted on the applicator cap and released by the hand wheel. It is made up of nitinol alloy which has the elasticity and memory that can restore its original shape after being released on the tissue. Then, the targeted lesion can be sutured to full-thickness by its teeth. Due to the different sizes of lesions, the OTSC has three sizes including 11, 12, and 14 mm. According to different indications, there are three different claw shapes such as blunt atraumatic type (a type), pointed traumatic edges (t type), and a special type for gastric wall closure (gc type). In this study, the 12 and 14 mm traumatic type (12/6t and 14/6t) were used in our institutions. Because the OTSC has the special occlusal structure, one advantage is the space between the teeth, which can prevent tissue necrosis by promoting blood supply. After installing the OTSC system and reaching the lesion, the twin grasper or anchor grasper is used to approximate the margins of the defect and pull the damaged tissue into the transparent applicator cap with the help of suction. When the targeted tissue is completely sucked into the cap, the hand wheel is turned to release the clip by tightening the thread. All the above procedures were performed by the experienced endoscopists who have completed formal training on the OTSC system in our Endoscopy Center.

### Statistical Analysis

We divided the patients into two groups according to whether OTSC was used. All of the baseline characteristics were analyzed, as shown in **Tables 3**, **4**. Propensity score matching was used to reduce selection bias of each group. Propensity score matching was estimated by using a multivariable logistic regression model with the following covariates: sex, age, lesion location, ulcer size, Forrest classification, antithrombotic therapy, hypertension, diabetes, and Blatchford score. All the categorical variables were reported as frequencies and percentages in the study and used to create a propensity score so as to match the “standard endoscopic therapy” group patients with the “OTSC” group. Patients were 1:1 matched using the nearest-neighbor method and with a caliper of 0.2. χ^2^ tests or Fisher's exact tests were used for all of the categorical variables. *P* < 0.05 were considered significant. R statistical software version 4.1.0 (www.r-project.org) and SPSS version 23.0 (IBM; Chicago, IL, USA) were used for the statistical analyses.

## Results

We retrospectively analyzed the collected clinical data. During this period, 80 patients [52 men and 28 women with an average age of 54 years (range 18–88 years)] treated with OTSC were divided into three categories based on indications, which were UNVGIB (*n* = 41), perforations (*n* = 34), and fistulas (*n* = 5). The characteristics of the patients are presented in Table 1. All patients were treated with only one traumatic version OTSC (Table 2).

**Table 1:** Overview on the characteristics and success rates of patients with different indications.

**Group**	**Patients (n)**	**Sex (male/female)**	**Median age (years) (min-max)**	**Technical success**	**Recurrence (n)**	**Clinical success**
Bleeding	41	35/6	55 (18–76)	41/41 (100%)	6	35/41 (85.4%)
Perforations	34	12/22	51 (19–88)	34/34 (100%)	0	34/34 (100%)
Fistulas	5	5/0	62 (55–71)	5/5 (100%)	1	4/5 (80.0%)
Total	80	52/28	54 (18–88)	80/80 (100%)	7	73/80 (91.3%)

**Table 2:** Indications and recurrence of OTSC placement.

**Indication**	***n***	**Type of clip (a/t/gc)**	**Technical success**	**Recurrence (*n*)**	**Clinical success**
**Bleeding**	41	(0/41/0)	41/41 (100%)	6	35/41 (85.4%)
Type					
Dieulafoy's lesion	21	(0/21/0)	21/21 (100%)	3	18/21 (85.7%)
Ulcer	19	(0/19/0)	19/19 (100%)	3	16/19 (84.2%)
Forrest Ia	5	(0/5/0)	5/5 (100%)	1	4/5 (80%)
Forrest Ib	6	(0/6/0)	6/6 (100%)	1	5/6 (83.3%)
Forrest IIa	8	(0/8/0)	8/8 (100%)	1	7/8 (87.5%)
Wound bleeding after EMR	1	(0/1/0)	1/1 (100%)	0	1/1 (100%)
Location					
Stomach	13	(0/13/0)	13/13 (100%)	2	11/13 (84.6%)
Duodenum	21	(0/21/0)	21/21 (100%)	3	18/21 (85.7%)
Remnant stomach after surgery	7	(0/7/0)	7/7 (100%)	1	6/7 (85.7%)
Blatchford score					
<6 (low risk)	2	(0/2/0)	2/2 (100%)	0	2/2 (100%)
≥6 (moderate and high risk)	39	(0/39/0)	39/39 (100%)	6	33/39 (84.6%)
Antithrombotic therapy					
Yes	7	(0/7/0)	7/7 (100%)	1	6/7 (85.7%)
No	34	(0/34/0)	34/34 (100%)	5	29/34 (85.3%)
**Perforations**	34	(0/34/0)	34/34 (100%)	0	34/34 (100%)
Duodenal ESD	12	(0/12/0)	12/12 (100%)	0	12/12 (100%)
Gastric ESD	20	(0/20/0)	20/20 (100%)	0	20/20 (100%)
Ileocecal EMR	1	(0/1/0)	1/1 (100%)	0	1/1 (100%)
Duodenal perforation	1	(0/1/0)	1/1 (100%)	0	1/1 (100%)
**Fistulas**	5	(0/5/0)	5/5 (100%)	1	4/5 (80%)
Tracheoesophageal fistula	1	(0/1/0)	1/1 (100%)	0	1/1 (100%)
Gastrobrochial fistula	1	(0/1/0)	1/1 (100%)	0	1/1 (100%)
Esophageal fistula	2	(0/2/0)	2/2 (100%)	1	1/2 (50%)
Duodenal fistula	1	(0/1/0)	1/1 (100%)	0	1/1 (100%)

*OTSC, over-the-scope clip; a, atraumatic; t, traumatic; gc, gastric wall closure type; EMR, endoscopic mucosal resection; ESD, endoscopic submucosal dissection*.

In general, the technical success was 80/80 (100.0%). The clinical success was 73/80 (91.3%), for six cases presented with recurrence of upper gastrointestinal bleeding and one patient failed in maintaining the OTSC before esophageal fistula healing. In addition, success was achieved in all perforated patients (Table 2).

### Upper Non-variceal Gastrointestinal Bleeding

All 41 patients with UNVGIB were treated with one OTSC respectively. In this group, the technical success was 41/41 (100.0%). However, six patients experienced rebleeding after OTSC placement. Therefore, the clinical success rate was 35/41 (85.4%).

According to the type of bleeding, lesions were divided into three types: “Dieulafoy's lesion,” “ulcer,” and “wound bleeding after EMR” (Table 2). Both technical success and clinical success were 100% in “wound bleeding after EMR.”

**Figure 2:**
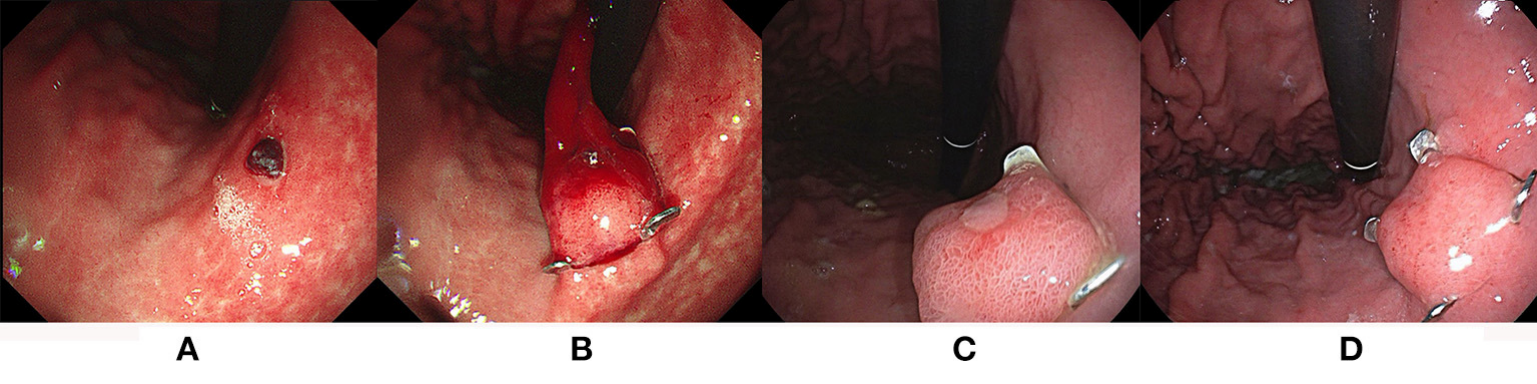
The OTSC application in the treatment of UNVGIB caused by Dieulafoy's lesion. **(A,B)** Dieulafoy's lesion was successfully treated with OTSC; **(C)** a follow-up endoscopy at 7 days; **(D)** a follow-up endoscopy at 2 months after OTSC placement confirmed clinical success.

In the Dieulafoy's lesion group of 21 patients, the technical success was 21/21 (100%). Nevertheless, three patients had rebleeding from the original exposed vessel and the clinical success was 18/21 (85.7%) (Figures 2A–D). The rebleeding occurred from 1 to 5 days. One rebleeding patient who had a Dieulafoy's lesion in the large diverticulum of descending duodenum underwent vascular interventional therapy after OTSC treatment, but the rebleeding was finally successfully stopped by tissue glue injection. One rebleeding patient accepted subsequent vascular interventional therapy, but the hemorrhage was eventually stopped by surgery. The last patient had a history of gastric cancer complicated with recurrence, refused any subsequent therapy, and eventually died.

For further evaluation of the hemostasis achieved using OTSC, we compared the OTSC treatment to standard endoscopic therapy in Dieulafoy's lesion (Table 3). In the unmatched cohort, 21 patients had OTSC placement, and 115 underwent standard endoscopic hemostasis. The OTSC group and the standard endoscopic therapy group differed with respect to antithrombotic therapy. Utilization of hemostasis with OTSC was more frequent in duodenum and antithrombotic therapy, whereas patients in the standard endoscopic therapy group were more frequently treated in the stomach. In order to mitigate the effects of baseline confounders, patients were matched into 20 pairs using propensity score matching. Covariates included in the model were sex, age, lesion location, antithrombotic therapy, comorbidities, and Blatchford score. In the matched cohort, rebleeding events were less common in the OTSC group (3/20, 15.0%) compared with the standard endoscopic therapy group (6/20, 30.0%), however, the rebleeding rates between the two groups were not significantly different (15.0 vs. 30.0%, *P* = 0.451).

**Table 3:** Clinical characteristics of the patients with Dieulafoy's lesion in the unmatched and matched cohorts.

**Variables**	**Unmatched cohort**			**Matched cohort**		
	**OTSC (*n* = 21)**	**Standard endoscopic therapy (*n* = 115)**	***p*-Value**	**OTSC (*n* = 20)**	**Standard endoscopic therapy (*n* = 20)**	***p*-Value**
Sex (male), *n* (%)	18 (85.7)	98 (85.2)	1.000	18 (90.0)	15 (75.0)	0.407
Age (≥60), *n* (%)	12 (57.1)	52 (45.2)	0.349	11 (55.0)	10 (50.0)	1.000
Location, *n* (%)			0.267			0.227
Stomach	11 (52.4)	80 (69.6)		11 (55.0)	15 (75.0)	
Duodenum	7 (33.3)	22 (19.1)		6 (30.0)	5 (25.0)	
Remnant stomach after surgery	3 (14.3)	13 (11.3)		3 (15.0)	0 (0.0)	
Antithrombotic therapy, *n* (%)	5 (23.8)	6 (5.2)	0.014	4 (20.0)	4 (20.0)	1.000
Hypertension, n (%)	5 (23.8)	19 (16.5)	0.532	5 (25.0)	4 (20.0)	1.000
Diabetes, n (%)	1 (4.8)	5 (4.3)	1.000	1 (5.0)	0 (0.0)	1.000
Blatchford score≥6, *n* (%)	21 (100.0)	107 (93.0)	0.609	20 (100.0)	17 (85.0)	0.231
Rebleeding, *n* (%)	3 (14.3)	28 (24.3)	0.405	3 (15.0)	6 (30.0)	0.451
Clinical success	85.7%	75.7%		85.0%	70.0%	

*OTSC, over-the-scope clip. Statistical analysis was performed with χ^2^ tests or Fisher's exact tests. p <0.05 were considered significant*.

There were 19 patients in the ulcer group, with 19/19 (100.0%) technical success. The clinical success of all 19 patients was 16/19 (84.2%). In terms of the characteristics of ulcers, Forrest Ia, Ib, and IIa each had one patient with recurrence of the original lesion, and the rebleeding time ranged from 1 to 4 days. The hemorrhages of three rebleeding patients were successfully stopped by endoscopic drug injection, interventional therapy, and surgical treatment, respectively. Depending on the site of bleeding lesion, the clinical success rate in the stomach was 11/13 (84.6%), in the duodenum was 18/21 (85.7%), and residual stomach after surgery was 6/7 (85.7%). In the assessment of the risk of rebleeding in all cases by Blatchford score, clinical success was 2/2 (100%) in two patients with low risk scores (<6 points), while 33/39 (84.6%) patients had moderate and high risk scores (≥6 points). Depending on antithrombotic therapy, both groups had acceptable clinical success (used 85.7%, unused 85.3%).

For further evaluation of the hemostasis achieved using OTSC, we compared OTSC treatment to standard endoscopic therapy in ulcer bleeding (Table 4). In the unmatched cohort, 19 patients had OTSC placement, and 380 underwent standard endoscopic therapy. The OTSC group and standard endoscopic therapy group differed with respect to lesion location and Forrest classification. Utilization of hemostasis with OTSC was more frequent in the remnant stomach after surgery, Forrest Ia, and antithrombotic therapy, whereas patients in the standard endoscopic therapy group had more lesions in the duodenum with Forrest IIb classification. Patients were matched into 17 pairs using propensity score matching, and the covariates included in the model were sex, age, site of bleeding, size of ulcer, Forrest classification, antithrombotic therapy, comorbidities, and Blatchford score. In the matched cohort, rebleeding events were less common in the OTSC group (3/17, 17.6%) compared with the standard endoscopic therapy group (5/17, 29.4%), however, the rebleeding rates between the two groups were not significantly different (17.6 vs. 29.4%, *P* = 0.688).

**Table 4:** Clinical characteristics of the patients with ulcers in the unmatched and matched cohorts.

**Variables**	**Unmatched cohort**			**Matched cohort**		
	**OTSC (*n* = 19)**	**Standard endoscopic therapy (*n* = 380)**	***p*-Value**	**OTSC (*n* = 17)**	**Standard endoscopic therapy (*n* = 17)**	***p*-Value**
Sex (male), *n* (%)	16 (84.2)	317 (83.4)	1.000	14 (82.4)	13 (76.5)	1.000
Age (≥60), *n* (%)	8 (42.1)	151 (39.7)	1.000	7 (41.2)	7 (41.2)	1.000
Location, *n* (%)			0.001			0.109
Stomach	2 (10.5)	80 (21.0)		1 (5.9)	5 (29.4)	
Duodenum	13 (68.4)	293 (77.1)		12 (70.6)	11 (64.7)	
Remnant stomach	4 (21.0)	7 (1.8)	0.157	4 (23.5)	1 (5.9)	
after surgery						
Size (≥1 cm), *n* (%)	11 (57.9)	155 (40.8)	0.157	11 (64.7)	9 (52.9)	0.728
Forrest, *n* (%)			0.000			0.224
Ia	5 (26.3)	23 (6.0)		4 (23.5)	3 (17.6)	
Ib	6 (31.6)	265 (69.7)		6 (35.3)	9 (52.9)	
IIa	8 (42.1)	71 (18.7)		7 (41.2)	3 (17.6)	
IIb	0 (0.0)	21 (5.5)		0 (0.0)	2 (11.8)	
Antithrombotic therapy, *n* (%)	3 (15.8)	28 (7.4)	0.176	1 (5.9)	2 (11.8)	1.000
Ulcer history, *n* (%)	5 (26.3)	72 (18.9)	0.384	5 (29.4)	6 (35.3)	1.000
Hypertension, *n* (%)	4 (21.0)	89 (23.4)	1.000	4 (23.5)	5 (29.4)	1.000
Diabetes, *n* (%)	4 (21.0)	34 (8.9)	0.095	4 (23.5)	3 (17.6)	1.000
Blatchford score≥6, *n* (%)	18 (94.7)	348 (91.6)	1.000	16 (94.1)	16 (94.1)	1.000
Rebleeding, *n* (%)	3 (15.8)	61 (16.0)	1.000	3 (17.6)	5 (29.4)	0.688
Clinical success	84.2%	83.9%		82.4%	70.6%	

*OTSC, over-the-scope clip. Statistical analysis was performed with χ^2^ tests or Fisher's exact tests. p <0.05 were considered significant*.

### Perforations

**Figure 3:**
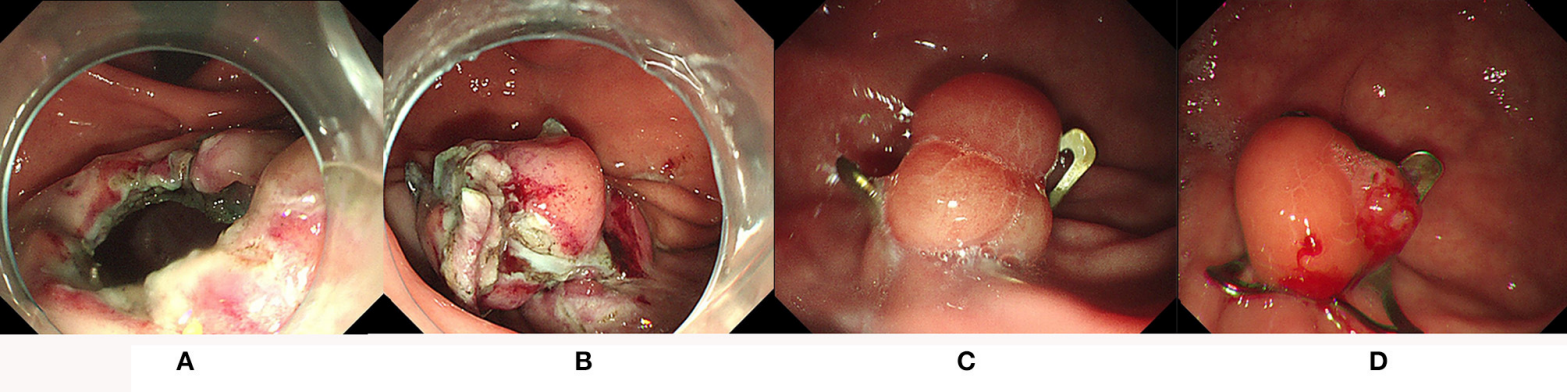
The iatrogenic perforation in the stomach was successfully closed by OTSC. **(A,B)** The perforation was successfully closed by OTSC; **(C)** a follow-up endoscopy at 6 months; **(D)** a follow-up endoscopy at 3 years showed the clip was still in place.

A total of 34 patients with gastrointestinal perforations were treated with OTSC successfully (Figures 3A–D). Among these patients, one case had an iatrogenic perforation opposite to the duodenal papilla during ERCP, and was sutured by OTSC successfully. The remaining 33 cases had iatrogenic perforations caused by ESD and EMR (32 ESD and 1 EMR). Most of the removed tumors were stromal tumors, as well as ectopic pancreas and leiomyomas, which were all underwent full-thickness resection. Before using OTSCs, some perforations were first closed by titanium clips and nylon cords but failed. A total of 20 cases were located in the stomach, 13 cases in the duodenum, and one case in the ileocecum. In brief, the OTSCs used for perforations both achieved 100% in technical and clinical success, regardless of etiology and location. During follow-up, there were no complications, such as re-perforation and bleeding.

### Fistulas

**Figure 4:**
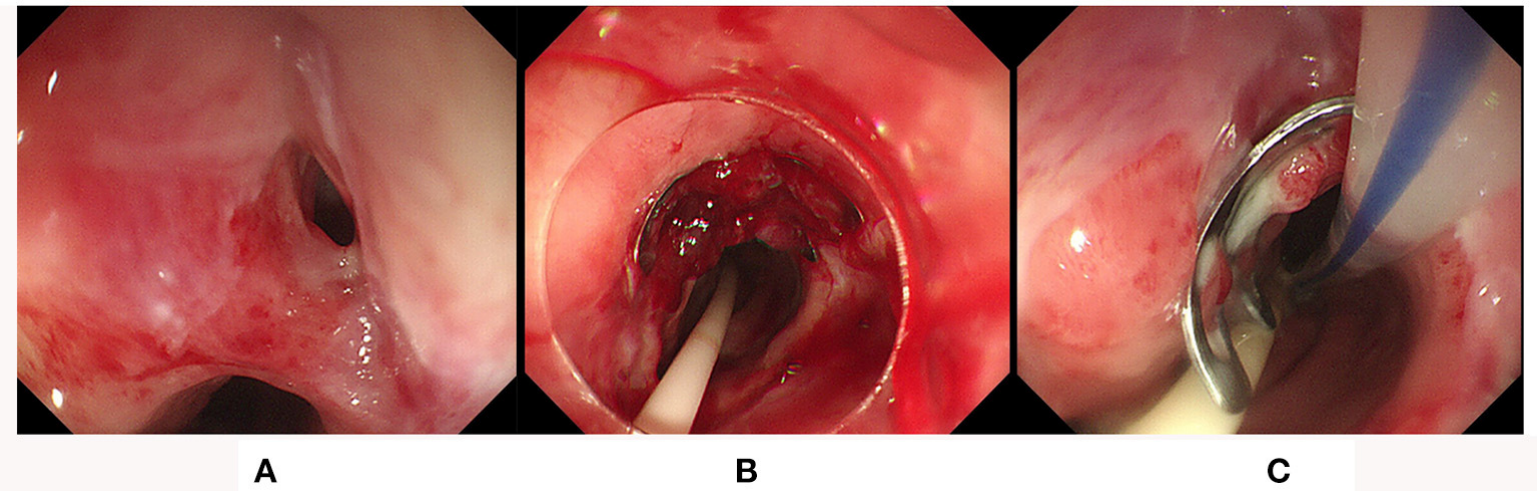
OTSC closure of the tracheoesophageal fistula. **(A,B)** The fistula was successfully treated by OTSC; **(C)** a follow-up endoscopy at 10 days after OTSC placement found the clip was still in place and the fistula was sealed successfully.

Five patients were treated with OTSCs due to fistulas, and each patient also used only one OTSC (Figures 4A–C). In this group of patients, the technical success was 5/5 (100%), and the clinical success was 4/5 (80%). The clinical failure patient had an esophageal fistula after thoracic surgery because of a foreign body in the esophagus. The fistula was successfully closed with one OTSC for the first treatment. However, the OTSC later failed when the patient had recurrent symptoms 1 month later. During the second treatment, a covered metallic stent was chosen to block the fistula which finally solved the issue. Another four patients had a tracheoesophageal fistula and gastrobrochial fistula after the surgery for esophageal cancer, a duodenal fistula after surgical repair of a duodenal bulb perforation, and an esophageal fistula caused by a foreign body in the esophagus, all of them were cured by OTSCs in one treatment.

## Discussion

To our knowledge, OTSC provides an effective and available alternative to manage various indications such as UNVGIB, perforations, and fistulas (20–25). However, clinical results were mainly from Europe, and there is a lack of data from East Asia, especially in China. Here, we evaluated a single-center experience about the application of OTSC in a Chinese endoscopy unit and also obtained favorable results for the use of OTSC. The overall technical and clinical success rates in our study were 100.0% (80/80) and 91.3% (73/80), respectively.

We found that OTSCs have achieved efficient and reliable hemostasis in UNVGIB, including Dieulafoy's lesion, ulcers, and other causes. Dieulafoy's lesion is characterized by a large submucosal artery without an associated ulcer (26). Identifying Dieulafoy's lesions could be challenging for the intermittent nature of bleeding and the absence of surrounding mucosal abnormalities. At present, endoscopic interventions are the main and primary treatment approach (27). However, the use of conventional endoscopic methods may be sometimes challenging because of the location of the lesion, size, and high risk of rebleeding, which may require angiographic embolization or on rare occasions surgical intervention for definitive hemostasis (27). At present, several studies reported only small cases on the use of OTSC for Dieulafoy's lesion, and achieved a reliable clinical effect (28–32). Our study reported 21 patients with Dieulafoy's lesion treated by OTSC, as far as we know, which was the current maximum sample size in published studies. The results indicated that rebleeding occurred in three patients (14.3%, 3/21) during the 30 day follow-up after successful hemostasis, and achieved a clinical success rate of 85.7% (18/21). So far, most studies on the use of OTSC in Dieulafoy's lesion include a limited sample size and lack a control group (16). Therefore, we tried to overcome this limitation by using propensity score analysis to balance the confounding factors between the OTSC and standard endoscopic therapy groups in our study. The result showed that the rebleeding rate of the standard endoscopic therapy group was twice as high as in the OTSC group (30.0 vs. 15.0%), however, the difference was not statistically significant. It is possible that the sample size was not large enough to be significant. To sum up, the OTSC could be raised as first-line treatment for Dieulafoy's lesion. Nevertheless, more large sample studies are required to prove its indications in clinical treatment.

At present, the studies found endoscopic treatment with OTSC to be superior to standard endoscopic therapy for patients with recurrent peptic ulcer bleeding, and was recommended in guidelines (15, 16, 33). However, the clinical data on OTSC treatment used as first-line therapy are limited to case series and retrospective studies (16, 33). Although there was one RCT evaluating the efficacy and safety of OTSC vs. standard endoscopic therapy for first-line treatment of UNVGIB recently published by Jensen et al. (29). However, methodological limitations to this study must be noted, including the relatively limited samples, and the inclusion of Dieulafoy's lesions in addition to peptic ulcers. In our study, we tried to compare the hemostasis achieved for ulcer bleeding between OTSC and standard endoscopic therapy groups by using propensity score analysis to balance the confounding factors. The result showed that the rebleeding rate of the standard endoscopic therapy group was almost twice as high as in the OTSC group (29.4 vs. 17.6%), however, the difference was not statistically significant. Similar to Dieulafoy's lesions, it is possible that the sample size was not large enough to be significant. Based upon current studies, the OTSC is shown to have an advantage over standard endoscopic therapy as first-line therapy for ulcer bleeding. Meanwhile, more large sample studies, especially randomized controlled studies, are required to evaluate its indications in clinical treatment as well.

Previous studies have shown that the risk of rebleeding of ulcers is significantly increased in patients who underwent antithrombotic therapy and have a relatively low success rate of hemostasis under the conventional endoscopic treatment (34–36). Improved endoscopic treatment of UNVGIB in anticoagulated patients might be achieved by new devices such as the OTSC, which allow for better tissue apposition and compression of bleeding vessels. For the time being, data on OTSC use for UNVGIB in patients under antithrombotic therapy are currently limited, quite different, and lack Chinese data. At present, only two studies reported a rebleeding rate from 9.5 to 38.5% among patients under antithrombotic therapy after OTSC treatment, and the rebleeding was more frequent among those who received antithrombotic therapy. However, the differences were not statistically significant (37, 38). In our study we found that the rebleeding between patients under antithrombotic therapy were similar with the patients without antithrombotic therapy (14.3 vs. 14.7%), which was consistent with the above results. The above indicates that OTSC may have an advantage in patients under antithrombotic therapy and more research is needed concerning the use of OTSC in anticoagulated patients.

In our study, there were six patients suffered from rebleeding under OTSC treatment, and the duration was from 1 to 5 days. In the subgroup, the duration of rebleeding was 1–5 days for Dieulafoy's lesion and 1–4 days for ulcers, which seems to be no difference. One clinical review reported that rebleeding typically occurs 1–4 days after initial conventional endoscopic therapy for Dieulafoy's lesion (39). For now, there are hardly any related studies that focus on the rebleeding time of Dieulafoy's lesion under OTSC treatment. However, whether OTSC treatment has an influence on the rebleeding time of Dieulafoy's lesion is not clear, and we will pay more attention to this in future.

With the development of endoscopic closure technology, it is rare that an iatrogenic perforation needs surgery. The effective treatment of OTSCs for iatrogenic perforations has been proposed in many previous studies (40–43). In general, the small iatrogenic perforation can be successfully closed by through-the-scope clips (TTSC) only (44). For large iatrogenic perforations, a nylon loop pouch suture has always been used. For this purpose, endoscopic closure of large procedure-related perforations using a single-channel endoscope was first proposed in our endoscopy center (45). In our study, the iatrogenic perforations were caused by EMR/ESD and ERCP, and were all closed by OTSC successfully. According to our follow-up, there was also no occurrence of delayed malignant events such as bleeding, perforation, and intestinal obstruction caused by the drop of the clip. Based on the 100% clinical success of iatrogenic perforation by OTSC and non-OTSC endoscopic therapy at the same period, we did not add counterparts for the treatment of OTSC in iatrogenic perforation.

Apart from gastrointestinal bleeding and perforations, OTSCs have been successfully used in fistulas, including tracheoesophageal, gastrobronchial, and esophageal fistulas (41, 46, 47). In our study, four fistulous patients were successfully treated with OTSCs while one developed recurrence during follow-up. The recurrent patient had an esophageal fistula after thoracic surgery because of a foreign body in the esophagus. Considering reasons for clinical failure, chronic fistulas lead to tissue necrosis and fibrosis around the fistula which makes it difficult for the clamp to bite, and insufficient nutritional supply after OTSC placement. Up to now, it is still difficult to manage GI fistulas with either endoscopic or surgical interventions and the optimal therapy is still being explored. Clinical success of the OTSC application in fistula management appears limited (14). Therefore, OTSC was rarely used in patients with fistulas in our endoscopic center and the sample size was only five. It is really difficult to conduct a comparative study based on so small a sample size, especially by using propensity score analysis, so we also did not add counterparts for the treatment of OTSC in patients with fistulas.

The OTSC is designed for full-thickness suture and is made of nickel-titanium alloy, which is the same material as cardiac and intracranial stents. Therefore, it can be worn for a long time and for life. During our follow-up in 80 patients, only one patient with a fistula experienced OTSC migration and resulted in symptom recurrence 1 month later. The OTSC was confirmed to be smoothly discharged from the body through X-ray, and there were no complications. Consistent with the previous reports, long-term OTSC attachment can be safe and effective, regardless of spontaneous detachment (21, 48).

However, our study still has the following limitations. On the one hand, the small number of related patients included, especially the closure of fistulas, may lead to a certain one-sidedness in the study results. On the other hand, as this was a single-center retrospective data analysis, the clinical data we selected were all from one tertiary care center of China, which could not avoid the inherent regional selection bias and represent the situation of other countries and hospitals. Therefore, larger sample and multi-center clinical studies are needed.

## Conclusions

In conclusion, our study confirms that the OTSC system plays a safe and effective role in UNVGIB, perforations, and fistulas as first-line treatment. However, for the clinical efficacy of OTSC treatment on some special lesions or patients, such as Dieulafoy's lesion or patients under antithrombotic therapy of UNVGIB, larger sample and multi-center experiences are eagerly needed.

## Data Availability Statement

The raw data supporting the conclusions of this article will be made available by the authors, without undue reservation.

## Ethics Statement

The studies involving human participants were reviewed and approved by the Ethics Committee of the First Affiliated Hospital of Nanchang University. The Ethics Committee waived the requirement of written informed consent for participation. All included cases were recorded in the Human Genetic Resources Center of the First Affiliated Hospital of Nanchang University.

## Author Contributions

JQ collected data, analyzed relevant information, and drafted the manuscript. JX collected the data and performed the data analysis. YZ contributed to figures collection and manuscript revision. FL contributed to statistical analysis. XP, ZZ, XS, and YC clinically managed the patients. XP designed the study, critically revised the paper, and approved the final submission. All authors contributed to the article and approved the submitted version.

## Funding

This work was supported by the Youth Science Foundation of Jiangxi Province (Grant No. 20192BAB215034).

## Conflict of Interest

The authors declare that the research was conducted in the absence of any commercial or financial relationships that could be construed as a potential conflict of interest.

## Publisher's Note

All claims expressed in this article are solely those of the authors and do not necessarily represent those of their affiliated organizations, or those of the publisher, the editors and the reviewers. Any product that may be evaluated in this article, or claim that may be made by its manufacturer, is not guaranteed or endorsed by the publisher.
